# Association of plasma level of high-mobility group box-1 with necroptosis and sepsis outcomes

**DOI:** 10.1038/s41598-021-88970-6

**Published:** 2021-05-04

**Authors:** Hongseok Yoo, Yunjoo Im, Ryoung-Eun Ko, Jin Young Lee, Junseon Park, Kyeongman Jeon

**Affiliations:** 1 Division of Pulmonary and Critical Care Medicine, Department of Medicine, Samsung Medical Center, Sungkyunkwan University School of Medicine, 81 Irwon-ro, Gangnam-gu, Seoul, 06351 Republic of Korea; 2 Department of Critical Care Medicine, Samsung Medical Center, Sungkyunkwan University School of Medicine, Seoul, Republic of Korea

**Keywords:** Predictive markers, Prognostic markers, Translational research

## Abstract

The role of high-mobility group box-1 (HMGB1) in outcome prediction in sepsis is controversial. Furthermore, its association with necroptosis, a programmed cell necrosis mechanism, is still unclear. The purpose of this study is to identify the association between the plasma levels of HMGB1 and the severity and clinical outcomes of sepsis, and to examine the correlation between HMGB1 and key executors of necroptosis including receptor-interacting kinase 3 (RIPK3) and mixed lineage kinase domain-like- (MLKL) proteins. Plasma HMGB1, RIPK3, and MLKL levels were measured with the enzyme-linked immunosorbent assay from the derivation cohort of 188 prospectively enrolled, critically-ill patients between April 2014 and December 2016, and from the validation cohort of 77 patients with sepsis between January 2017 and January 2019. In the derivation cohort, the plasma HMGB1 levels of the control (n = 46, 24.5%), sepsis (n = 58, 30.9%), and septic shock (n = 84, 44.7%) groups were significantly increased (P < 0.001). A difference in mortality between high (≥ 5.9 ng/mL) and low (< 5.9 ng/mL) HMGB1 levels was observed up to 90 days (Log-rank test, *P* = 0.009). There were positive linear correlations of plasma HMGB1 with RIPK3 (R^2^ = 0.61, *P* < 0.001) and MLKL (R^2^ = 0.7890, P < 0.001). The difference in mortality and correlation of HMGB1 levels with RIPK3 and MLKL were confirmed in the validation cohort. Plasma levels of HMGB1 were associated with the severity and mortality attributed to sepsis. They were correlated with RIPK3 and MLKL, thus suggesting an association of HMGB1 with necroptosis.

## Introduction

Sepsis is a severe and fatal disease characterized by a dysregulated host response to infection causing organ failure and death^1^. Owing to its complex nature, the pathogenesis of sepsis is not yet fully comprehended. Thus, the efforts expended in the development of cure have been unsuccessful^2^. In addition, the current definition of sepsis identifies a heterogeneous population of individuals with diverse patterns of immune response, organ dysfunction, and clinical outcomes^1^. Therefore, early diagnosis, precise stratification of severity, and accurate outcome predictions are crucial in managing patients with sepsis^3^. However, to-this-date, no biomarkers have been proven sufficient to meet the needs of clinicians in clinical practice^4^.

The high-mobility group box-1 (HMGB1) is a nonhistone, chromatin-associated nuclear protein originally described to function as a factor for the regulation of gene expression and transcription^5^, and is one of the well-known proteins of damage-associated molecular patterns (DAMPs) that activate the immune system that initiates systemic inflammation cascade^6,7^. In sepsis, HMGB1 not only triggers neutrophil recruitment but also induces macrophages and endothelial cell stimulation for proinflammatory cytokine production^7^. Animal studies have shown that inhibition of HMGB1 in septic models improves survival^8,9^. Furthermore, elevated levels of HGMB1 in sepsis patients have been reported consistently^10,11^. However, the role of HMGB1 as a sepsis biomarker in the assessment of severity and prediction of mortality is still controversial^12^. In addition, growing evidence indicates that among various mechanisms, HMGB1 leakage may be induced by the regulated cell death (RCD) mechanism of necroptosis, caspase-independent receptor-interacting protein kinase (RIPK), and mixed lineage kinase domain-like protein (MLKL)^13^. However, the association of the plasma levels of HMGB1 with necroptosis in sepsis has not been investigated.

The objective of our study is to investigate the association between the plasma levels of HMGB1 and the severity and outcomes of sepsis. In addition, the plasma levels of HMGB1 were correlated to those of RIPK3 and MLKL to identify the relationship of HMGB1 with necroptosis^14,15^.

## Methods

This study analyzed data of critically-ill adult patients enrolled in the prospective cohort of Samsung Medical Center Registry of Critical Illness (SMC-RoCI). SMC-RoCI is an active registry that started enrolling patients in April 2014. Data of the cohort and details of the study protocols have been published previously^16,17^. In brief, critically-ill adult patients aged 19 years or older admitted to the medical ICU were considered eligible for inclusion in the registry. Exclusion criteria included ICU admission for a simple procedure or postsurgical care, end-of-life decision, discharge within 24 h of admission, and persistent bleeding or hemoglobin < 8 g/dL which limits serial blood collection. This study was approved by the institutional review board of the Samsung Medical Center and performed in compliance with Helsinki declaration. Written informed consent was obtained from patients or their legally authorized representative prior to enrollment.

### Study population

**Figure 1:**
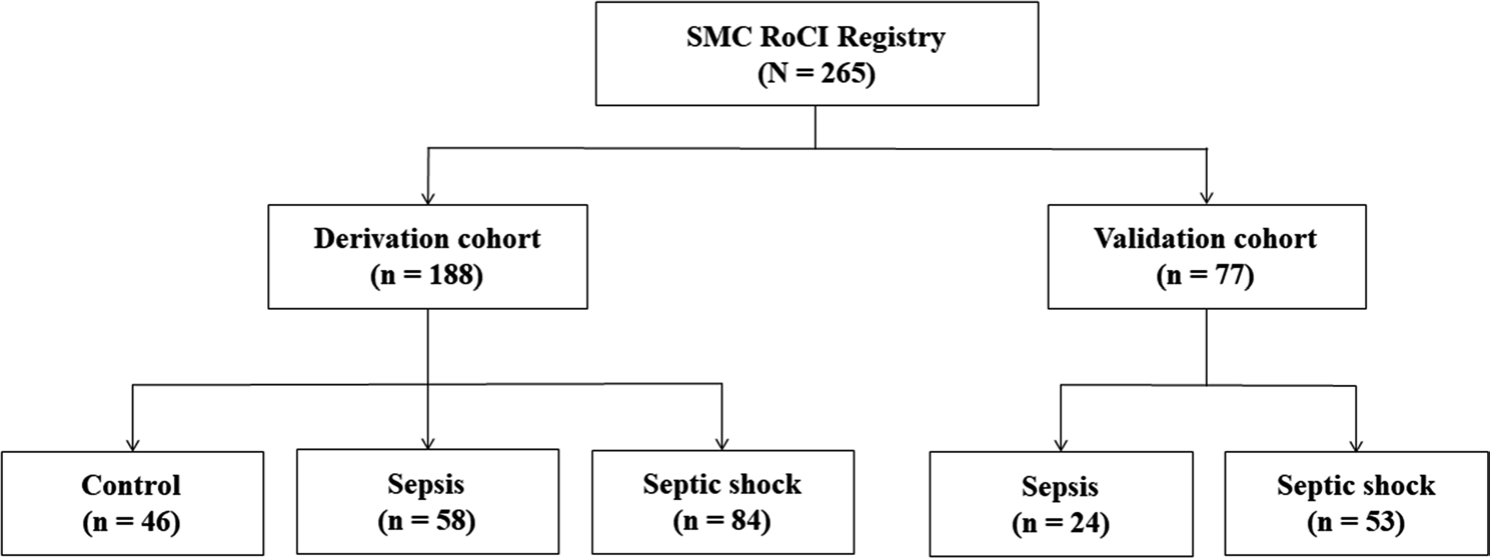
Flow diagram of studied patients (SMC-RoCI, Samsung Medical Center Registry of Critical Illness).

Adult, critically-ill patients registered in SMC-RoCI admitted to the medical intensive care unit (ICU) of Samsung Medical Center (1,989 beds, university affiliated, tertiary referral hospital in Seoul, South Korea), were included in our study. One-hundred eighty-eight critically-ill patients who were enrolled between April 2014 and December 2016 were analyzed. Seventy-seven patients with sepsis registered between January 2017 and January 2019 were used as the validation cohort (Fig. 1).

### Data collections

Clinical data, including patient demographics, reason for ICU admission, laboratory data, and severity of illness scores were collected by a trained research coordinator at the time of enrollment using the electronic health records. The sequential organ failure assessment (SOFA)^18^, simplified acute physiology score 3 (SAPS 3)^19^, and acute physiology and chronic health evaluation II (APACHE II) scores^20^ were used to evaluate the disease severity. The primary outcome measure was the 28-day mortality. In-hospital mortality and 90-day mortality were measured as secondary outcomes.

The third International Consensus Definitions for Sepsis and Septic Shock (Sepsis-3) was used to diagnose sepsis and septic shock^1^. Patients enrolled before the publication of Sepsis-3 were reviewed and re-categorized according to the new definition. The control group was defined as the patient group that did not meet the Sepsis-3 criteria.

### Measurement of plasma HMGB1, RIPK3, and MLKL

In accordance with the purpose of the SMC-RoCI registry, the study protocol requires the collection of 19 mL whole blood samples from all the patients within 48 h of ICU admission. Centrifuged samples were stored at −80 °C for future use. The details of the study protocol and sample process are available from a previously published study^16^. Plasma HMGB1, RIPK3, and MLKL levels were measured with the enzyme-linked immunosorbent assay with a commercially available kit according to the manufacturer’s manual (HMGB1, IBL International, Hamburg, Germany; RIPK3, CUSABIO, Wuhan, China; MLKL, LifeSpan BioSciences, Seattle, USA)^16,21,22^.

### Statistical analysis

All data are reported as numbers (percentages) for categorical variables and as medians with interquartile ranges (IQR, 25^th^–75^th^ percentiles) for continuous variables. We used the chi-squared test or Fisher’s exact test to compare categorical variables, and the Mann–Whitney *U* test to compare continuous variables. The differences in HMGB1 level among sepsis, septic shock, and control groups were analyzed by the Kruskal–Wallis test. The patients with sepsis were divided into high- and low-plasma level groups of HMGB1 according to the best cut-off level determined by Youden’s index for the prediction of the 28-day mortality^23^. We used the log-rank test to compare the differences in the 90-day survival. The correlation analyses of HMGB1 with RIPK3 and MLKL were performed with linear regression based on the Pearson’s correlation and Spearman’s rank correlation coefficients.

Statistical analysis was performed using SPSS (version 20.0, IBM, Chicago, IL, USA), and P < 0.05 was considered statistically significant.

## Results

The baseline characteristics of the derivation cohort of 188 critically-ill patients are summarized in Table 1. The study population consisted of 58 (30.9%) sepsis, 84 (44.7%) septic shock, and 46 (24.5%) control patients. One-hundred fifteen (61.2%) patients required vasopressor support upon ICU admission. The median scores of SAPS 3, APACHE II, and SOFA were 52 (44–59), 23 (18–8), and 8 (5–11), respectively. The interval between ICU admission to blood sampling was 30 (21–37) hours.

**Table 1:** Baseline characteristics of the 188 patients who participated in the derivation cohort.

Characteristics	No. of patients (%) or median (IQR)
**Age, years**	64 (54–73)
**Gender, male**	120 (63.8)
**Co-morbidities**	
Cancer	92 (48.9)
Solid tumor	61 (32.4)
Hematologic malignancy	31 (16.5)
Diabetes	55 (29.3)
Chronic obstructive pulmonary disease	18 (9.6)
Chronic kidney disease	15 (8.0)
Myocardial infarction	11 (5.9)
Congestive heart failure	9 (4.7)
Cerebrovascular disease	6 (3.2)
Chronic liver disease	1 (0.5)
**Charlson comorbidity index**	2 (1–3)
**Clinical status on ICU admission**	
Need for MV	95 (50.5)
Need for vasopressor support	115 (61.2)
**Laboratory findings**	
PaO_2_/FiO_2_ ratio	193.3 (125.7–300.0)
CRP, mg/dL	10.1 (3.2–19.0)
Lactic acid, mg/dL	2.7 (1.7–4.1)
**Severity of illness**	
SAPS 3	52 (44–59)
APACHE II score	23 (18–8)
Initial SOFA score	8 (5–11)
**Plasma HMGB1, ng/mL**	3.5 (1.8–6.2)
**Outcome**	
7-day mortality	15 (8.0)
28-day mortality	41 (21.8)
90-day mortality	59 (31.4)
In-hospital mortality	48 (25.5)

APACHE II, acute physiology and chronic health evaluation II; CRP, C-reactive protein; HMGB1, high-mobility group box-1; ICU, intensive care unit; IQR, interquartile range; MV, mechanical ventilation; SAPS 3, simplified acute physiology score 3; SOFA, sequential organ failure assessment.

**Figure 2:**
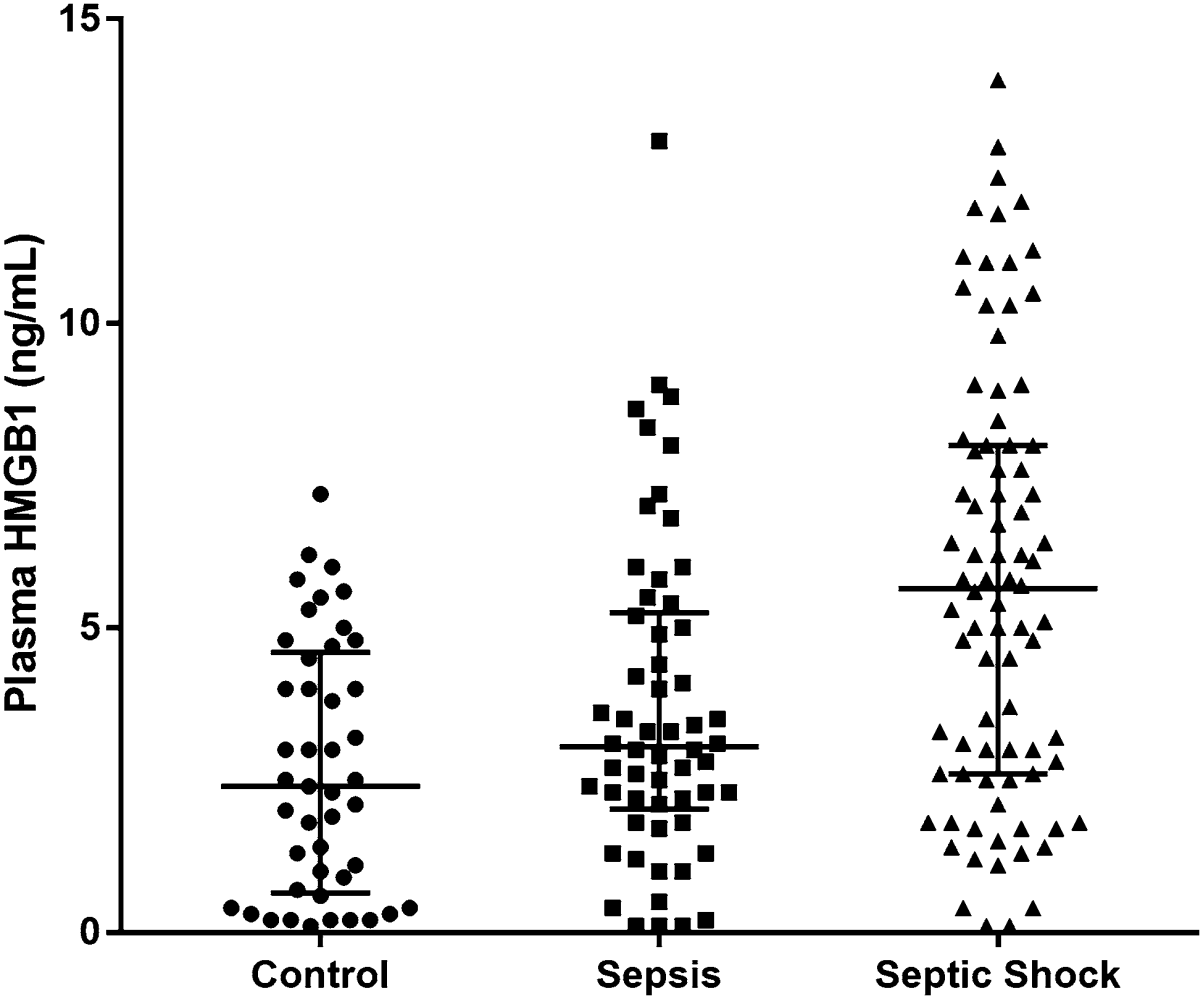
Plasma levels of high-mobility group box-1 (HMGB1) in control, sepsis, and septic shock in the derivation cohort (n = 188) (*P* for trend < 0.001). The bars represent median and interquartile ranges.

**Figure 3:**
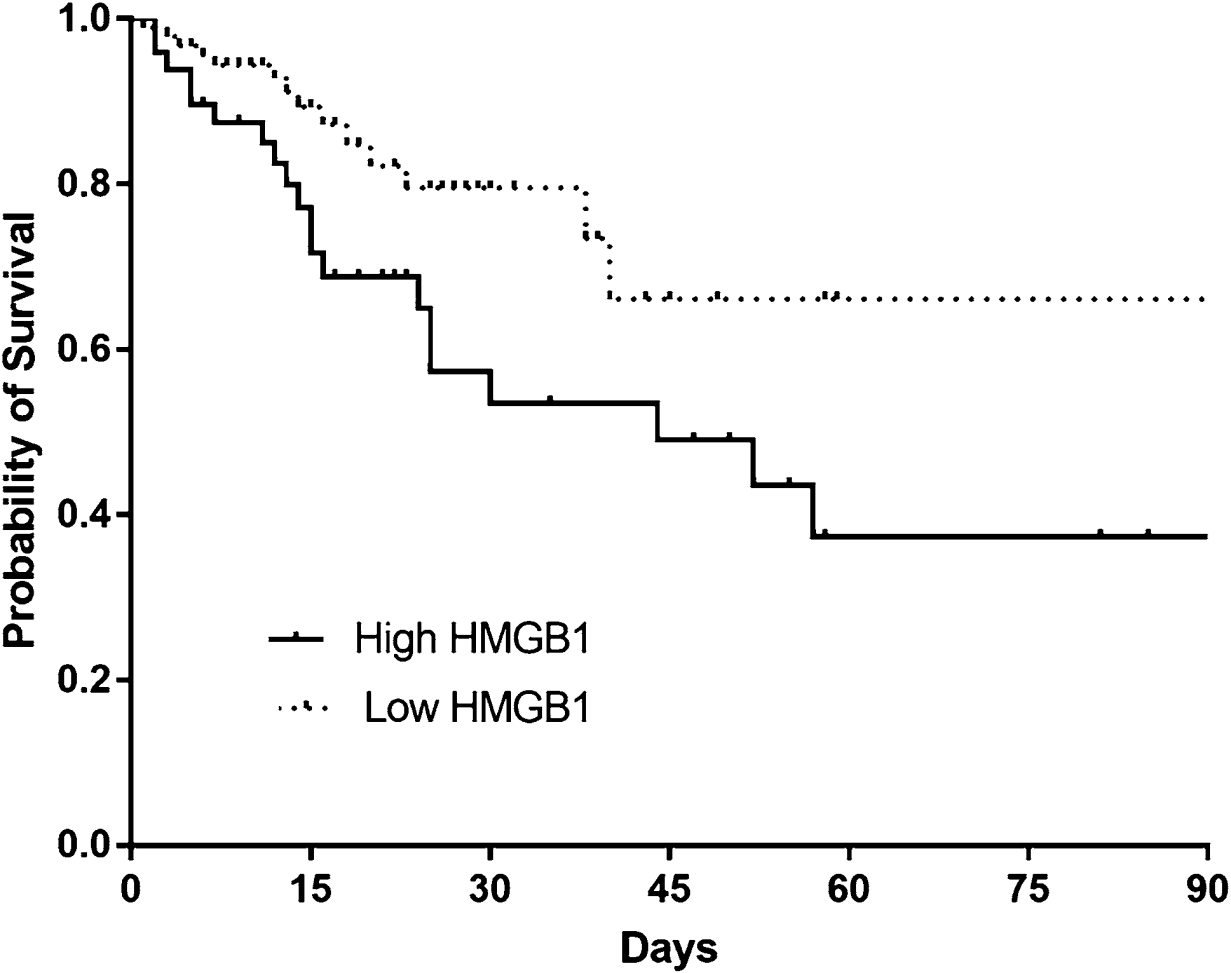
Kaplan–Meier survival analysis comparing sepsis and septic shock patients with high- and low-plasma levels of HMGB1 from the derivation cohort (n = 142) (*P* = 0.009, log-rank test).

The median levels of HMGB1 for the control, sepsis, and septic shock groups were 2.4 (0.6–4.6) ng/mL, 3.1 (2.0–5.3) ng/mL, and 5.7 (2.6–8.0) ng/mL, respectively. These values demonstrated a statistically significant trend of increase (*P* for trend < 0.001) (Fig. 2). The optimal cut-off level of HMGB1 for predicting the 28-day mortality in 142 sepsis patients was 5.9 ng/mL. Comparisons of clinical characteristics and mortality of sepsis patients between high (≥ 5.9 ng/mL, n = 49) and low (< 5.9 ng/mL, n = 93) plasma levels of HMGB1 are listed in Table 2. The number of patients who required vasopressor support, blood lactic acid levels, the number of septic shock patients, and severity of illness based on SAPS 3, APACHE II, and SOFA scores, was higher in the case of the high-HMGB1 group. Furthermore, the 28-day, in-hospital, and 90-day mortalities were significantly higher in the high-HMGB1 group. The Kaplan–Meier survival analysis depicted a significant difference in 90-day survival (*P* = 0.009) (Fig. 3).

**Table 2:** Characteristics of patients with sepsis stratified according to the best cut-off level of plasma HMGB1 from the derivation cohort (n = 142).

	Low HMGB1 (n = 93)	High HMGB1 (n = 49)	*P* value
**Age, years**	65 (54–74)	67 (51–72)	0.828
**Gender, male**	64 (68.8)	31 (63.3)	0.504
**Co-morbidities**			
Cancer	46 (49.5)	28 (57.1)	0.384
Solid tumor	30 (32.3)	20 (40.8)	0.310
Hematologic malignancy	16 (17.2)	8 (16.3)	0.894
Diabetes	28 (30.1)	15 (30.6)	0.950
Chronic kidney disease	6 (6.5)	5 (10.2)	0.513
Myocardial infarction	4 (4.3)	3 (6.1)	0.693
Congestive heart failure	1 (1.1)	4 (8.2)	0.048
Cerebrovascular disease	3 (3.2)	3 (6.1)	0.415
**Charlson comorbidity index**	2 (1–3)	2 (1–3)	0.244
Septic shock	46 (49.5)	38 (77.6)	0.001
**Clinical status on ICU admission**			
Need for MV	38 (40.9)	26 (53.1)	0.165
Need for vasopressor support	59 (63.4)	41 (83.7)	0.012
**Laboratory findings**			
PaO_2_/FiO_2_ ratio	196.0 (128.8–285.7)	167.0 (123.5–272.3)	0.191
CRP, mg/dL	12.0 (3.4–20.1)	15.8 (6.5–26.1)	0.037
Lactic acid, mg/dL	2.3 (1.7–3.3)	3.9 (2.3–5.7)	< 0.001
**Severity of illness**			
SAPS 3	51 (44–58)	56 (50–74)	0.004
APACHE II score	21 (17–26)	28 (21–25)	< 0.001
Initial SOFA score	7 (6–10)	11 (8–13)	< 0.001
**Outcome**			
7-day mortality	5 (5.4)	7 (14.3)	0.109
28-day mortality	13 (14.0)	17 (34.7)	0.004
90-day mortality	23 (24.7)	25 (51.0)	0.002
In-hospital mortality	14 (15.1)	22 (44.9)	< 0.001

APACHE II, acute physiology and chronic health evaluation II; CRP, C-reactive protein; ICU, intensive care unit; MV, mechanical ventilation; PaO_2_/FiO_2_, arterial partial pressure of oxygen/fraction of inspired oxygen; SAPS 3, simplified acute physiology score 3; SOFA, sequential organ failure assessment.

**Figure 4:**
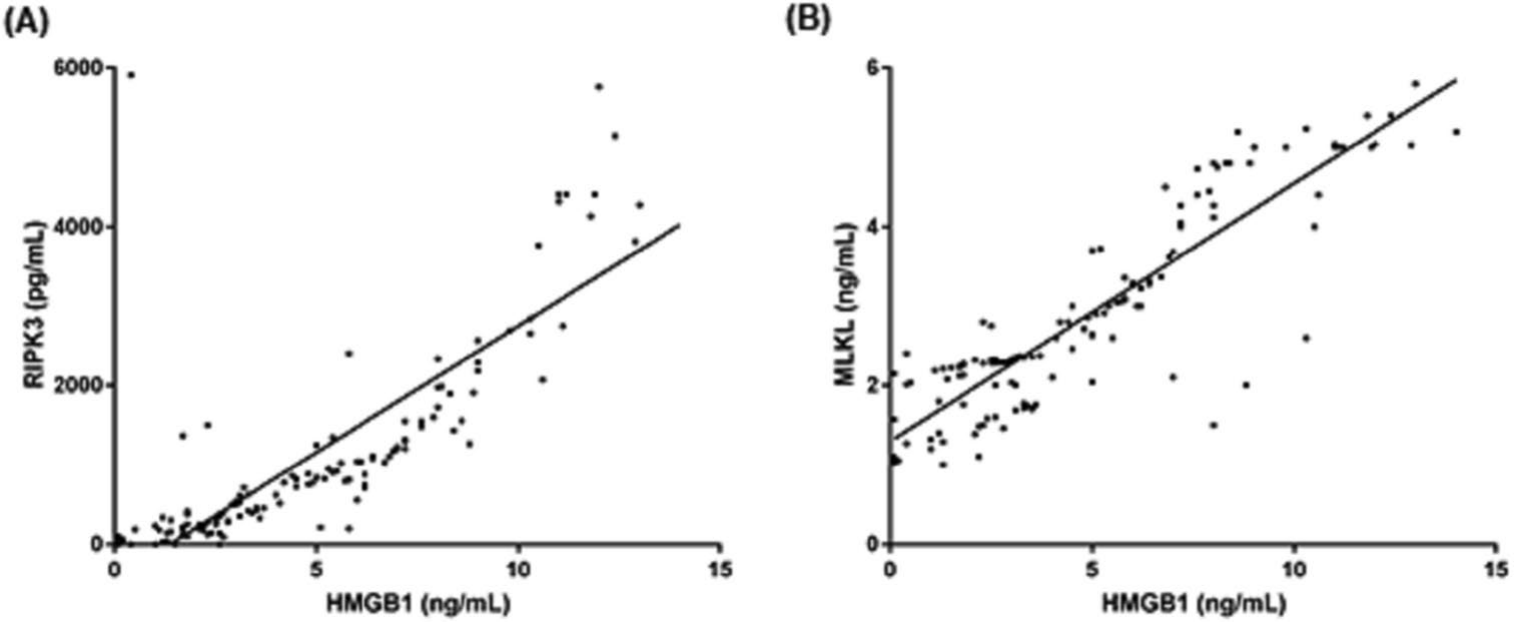
Correlation between plasma levels of HMGB1 and receptor-interacting kinase 3 (RIPK3) (A) and mixed lineage-like-domain protein (MLKL) (B) in patients with sepsis from the derivation cohort (n = 142) [RIPK3; slope 318.5 (95% confidence interval (CI): 279.6–357.4), R^2^: 0.6156 (*P* < 0.001), Pearson’s: 0.807 (*P* < 0.001), Spearman’s rho: 0.885 (*P* < 0.001)] [MLKL; slope = 0.3251 (95% CI: 0.2790–0.3532), R^2^: 0.7890 (*P* < 0.001), Pearson’s: 0.888 (*P* < 0.001), Spearman’s rho: 0.852 (*P* < 0.001)].

There was a positive linear association between the plasma levels of HMGB1 and RIPK3 (Pearson’s r = 0.807, Spearman’s rho = 0.885, R^2^ = 0.6516, *P* < 0.001) (Fig. 4A). In the same manner, the plasma levels of HMGB1 and MLKL demonstrated a positive linear relationship (Pearson’s r = 0.852, Spearman’s rho = 0.888, R^2^ = 0.789, *P* < 0.001) (Fig. 4B).

**Figure 5:**
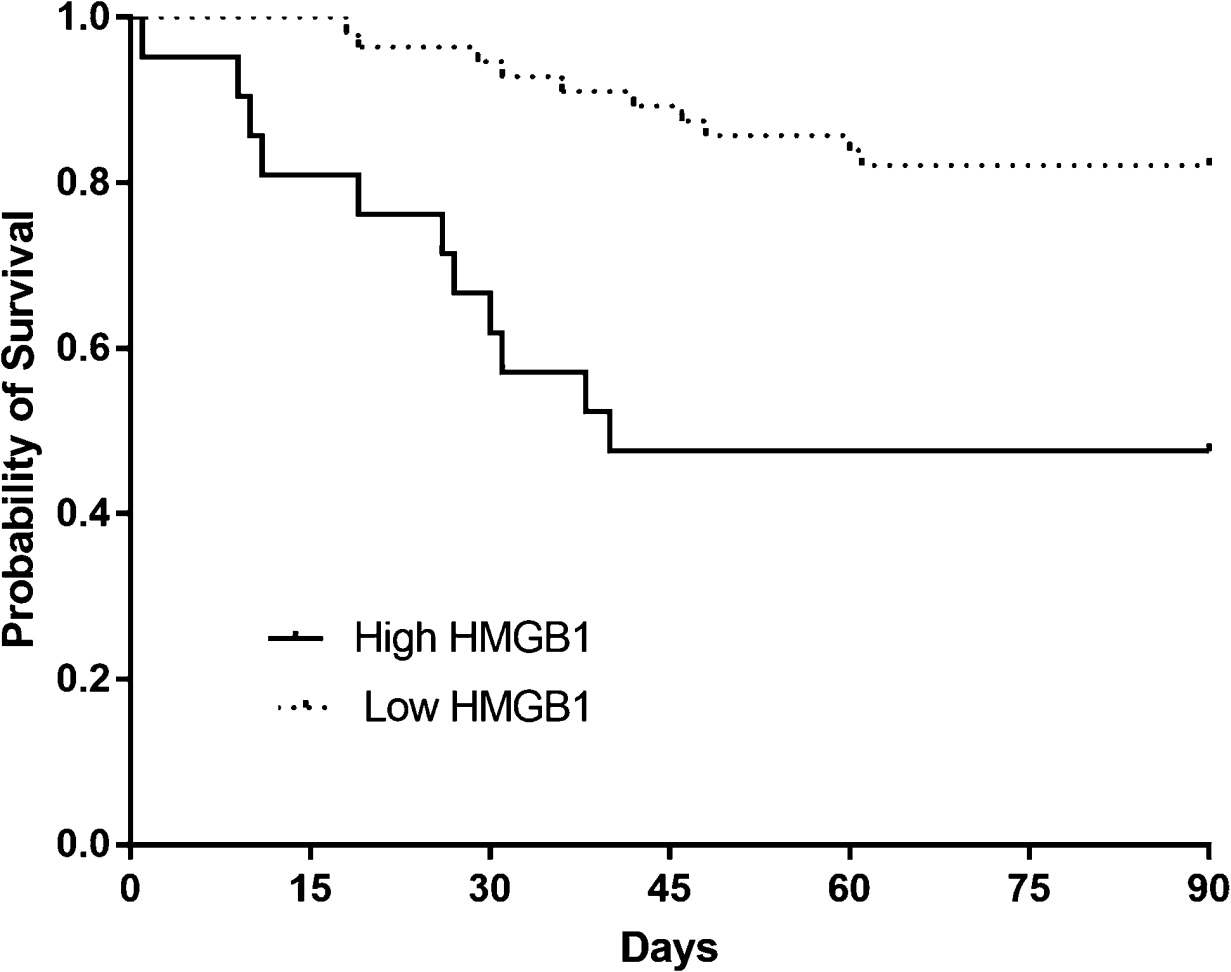
Kaplan–Meier survival analysis comparing patients of validation cohort with high- and low-plasma levels of HMGB1 (n = 77) (*P* < 0.001, log-rank test).

Validation cohort of 77 patients consisted of 24 (31.2%) patients with sepsis and 53 (68.8%) patients with septic shock. The plasma levels of HMGB1 in patients with septic shock were significantly higher than those in sepsis patients [4.0 ng/mL (2.4–5.8) vs. 5.0 ng/mL (4.0–6.5), *P* = 0.023]. The validation cohort was then classified into the groups of high (n = 21) and low (n = 56) HMGB1 levels according to the optimal cut-off level of 5.9 ng/mL determined from the derivation cohort (Table 3). Although the baseline characteristics of age, gender, co-morbidities, clinical status on ICU admission, or severity of illness scores were similar in all the groups, the 28-day mortality was higher in the high-HMGB1 group (33.3% vs. 3.6%, *P* < 0.001). In addition, in-hospital and 90-day mortalities significantly differed between the two groups (Table 3, Fig. 5). The positive correlations of plasma HMGB1 levels with those of RIPK3 (R^2^ = 0.7394, *P* < 0.001) and MLKL (R^2^ = 0.584, *P* < 0.001) were also observed in the validation cohort (see Supplementary Figures).

**Table 3:** Characteristics of patients with sepsis stratified according to the level of plasma HMGB1 from the validation cohort (n = 77).

Characteristics	Overall		Low HMGB1 (n = 56)		High HMGB1 (n = 21)	*P* value
**Age, years**	67 (59–75)		69 (55–76)		66 (63–71)	0.792
**Gender, male**	54 (70.1)		38 (67.9)		16 (76.2)	0.477
**Co-morbidities**						
Cancer	43 (55.8)		29 (51.8)		14 (66.7)	0.242
Solid tumor	32 (41.6)		21 (37.5)		11 (52.4)	0.238
Hematologic malignancy	12 (15.6)		9 (16.1)		3 (14.3)	1.000
Diabetes	26 (33.8)		18 (32.1)		8 (38.1)	0.623
Chronic kidney disease	7 (9.1)		5 (8.9)		2 (9.5)	1.000
Myocardial infarction	4 (5.2)		2 (3.6)		2 (9.5)	0.298
Congestive heart failure	3 (3.9)		3 (5.4)		0	0.558
Cerebrovascular diseases	4 (5.2)		4 (7.1)		0	0.570
**Charlson cormobidity index**	2 (1–3)		2 (1–3)		2 (1–3)	0.751
Septic shock	53 (68.8)		37 (66.1)		16 (76.2)	0.393
**Clinical status on ICU admission**						
Need for mechanical ventilation	34 (44.2)		24 (42.9)		10 (47.6)	0.708
Need for vasopressor support	62 (80.5)		46 (82.1)		16 (76.2)	0.537
**Laboratory findings**						
PaO_2_/FiO_2_ ratio	233.8 (150.7–351.9)		222.7 (149.5–306.9)		302.5 (162.7–386.2)	0.153
CRP, mg/dL	13.1 (6.9–25.6)		12.2 (5.4–23.9)		15.4 (8.1–31.0)	0.088
Lactic acid, mg/dL	3.0 (1.9–4.6)		2.7 (1.7–4.2)		3.8 (2.2–4.9)	0.098
**Severity of illness**						
SAPS 3	57 (48–65)		57 (47–63)		60 (54–66)	0.212
APACHE II score	25 (20–30)		25 (18–28)		29 (21–32)	0.084
Initial SOFA score	9 (7–11)		9 (7–11)		10 (8–12)	0.052
7-day mortality	1 (1.3)		0		1 (4.8)	0.273
28-day mortality	9 (11.7)		2 (3.6)		7 (33.3)	0.001
90-day mortality	21 (27.3)		10 (17.9)		11 (52.4)	0.002
In-hospital mortality	17 (22.1)		7 (12.5)		10 (47.6)	0.002

APACHE II, acute physiology and chronic health evaluation II; CRP, C-reactive protein; ICU, intensive care unit; PaO_2_/FiO_2_, arterial partial pressure of oxygen/fraction of inspired oxygen; SAPS 3, simplified acute physiology score 3; SOFA, sequential organ failure assessment.

## Discussion

Based on data analyses from 188 critically-ill patients and additional 77 patients with sepsis from the prospective SMC-RoCI registry, we observed that the plasma levels of HMGB1 were associated with the severity and mortality of sepsis. In addition, plasma levels of HMGB1 showed a positive linear relationship with those of RIPK3 and MLKL, key executors of RCD mechanism of necroptosis. The results of our study suggest that plasma HMGB1 may be a potential biomarker for severity assessment and mortality prediction of sepsis. Furthermore, the study and its outcomes suggest that necroptosis is likely a major source of plasma HMGB1 in sepsis.

The results of previous studies on the relationship between plasma HMGB1 and sepsis mortality are contradictory. Huang et al.^24^ and Ueno et al.^25^ demonstrated that high-plasma HMGB1 levels were associated with higher mortality in patients affected with sepsis and in septic shock patients who underwent polymyxin B hemoperfusion. However, other studies observed a negative relationship between the HMGB1 levels and prognosis^10,26,27^. Recent systematic review and meta-analysis of eight studies on sepsis patients found marginal differences in HMGB1 levels between survivors and nonsurvivors (pooled mean difference 1.2 ng/mL; 95% confidence interval (CI) 0.0–2.4; *P* = 0.05) ^12^. In our study, a significant difference in mortality between patients with high- and low-HMGB1 levels determined by Youden’s index was observed up to 90 days.

There are several possibilities for these conflicting results. First, a difference should be recognized in the statistical method used in our study compared with the previous studies. Unlike previous studies that compared plasma HMGB1 levels between survivors and nonsurvivors, we calculated Youden’s index first to obtain the best cut-off point for the prediction of the 28-day mortality, and then compared the mortalities between the groups. Considering that there are certain advantages and disadvantages among statistical methods in evaluating the performance of biomarkers, we decided to calculate the optimal cut-off level to identify the discriminatory power as well as the clinical applicability of HMGB1 in sepsis rather than compare the levels between survivors and nonsurvivors^28,29^. Furthermore, we confirmed the result in the validation cohort using the same cut-off level from the derivation cohort. Another factor, in addition to the statistical difference that needs to be considered pertains to the chronological changes of the HMGB1 levels. In their study of 42 patients with septic shock, Gibot et al. noticed increases in HMGB1 levels between days 1 and 3 among nonsurvivors, whereas a progressive but statistically nonsignificant decrease was noted in HMGB1 levels among survivors^30^. In the same study, the level of HMGB1 at day 3 (and not that at day 0) was associated with increased mortality. Given the fact that HMGB1 is a late mediator of sepsis^31,32^, this result concurs with several studies advocating that delayed measurement of serial changes in HMGB1 may be predictive of mortality^11,33^. Moreover, in a study of sepsis patients with various sources of infection, the kinetics of HMGB1 release showed different patterns according to the primary source of infection^26^. These findings suggest that the timing of the sampling and source of infection may affect the significance of HMGB1 in the prediction of mortality owing to sepsis. However, we cannot confirm whether this is the reason for the conflicting results of the previous studies. In fact, withdrawal of blood was performed within 24 h of ICU admission in our study which may not reflect the delayed phase of HMGB1 elevation. In addition, late elevation was regarded as a consequence of active release from immune cells, while immediate surge may occur from cell damage or necrosis. Therefore, elevated HMGB1 in our cohort may by a manifestation of cell death. Given that our knowledge on the origin of HMGB1 in sepsis is limited, we are not able to draw inferences with certainty at this time. Additional studies identifying the kinetics of plasma HMGB1 are essential to better understand the role of HMGB1 as a biomarker and its meaning in the pathogenesis of sepsis. Finally, heterogeneity of sepsis may have contributed to the discordance. In a recent study on multiple observational cohorts of sepsis, Seymour et al. classified sepsis patients into novel phenotypes based on clinical and laboratory variables^34^. Each phenotype demonstrated distinct patterns of organ failure and clinical outcomes as well as distributions of biomarkers. Disparity in proportion of specific subtypes of sepsis patients among previous studies and ours have resulted in conflicting significance of HMGB1 in mortality prediction. Further research on the association of HMGB1 and sepsis phenotypes is necessary to determine the utility of HMGB1 as a biomarker.

One of the most notable findings of our study is the strong association of plasma levels of HMGB1 with those of RIPK3 and MLKL, the key mediators of necroptosis^14,15^. HMGB1 can be actively secreted by immune cells, such as macrophages, dendritic cells, or monocytes in a delayed manner (> 6 h) as well as passively released through necrosis or RCDs other than apoptosis in an immediate manner^35^. Nevertheless, the source of extracellular release of HMGB1 is not yet clearly confirmed in sepsis. In vitro studies and animal models show a delayed surge of HMGB1 after lipopolysaccharide or endotoxin stimulation which suggests active secretion from immune cells as one of the mechanisms of HMGB1 release in sepsis^32,36^. By contrast, a few recent studies have reported that HMGB1 is released from caspase-independent necrotic-like cell deaths of macrophages and myoblasts, respectively^37,38^. The results support the hypothesis of necroptosis as a mechanism of an HMGB1 shift in sepsis. However, its association with RIPK1/RIPK3-dependent necroptosis has not been investigated. To our knowledge, we have demonstrated, for the first time, the association of plasma levels of surrogate markers of necroptosis activity RIPK3 and MLKL, with that of HMGB1 in patients with sepsis. The strong correlation presents compelling evidence that the early rise of HMGB1 may be mainly attributed to necroptosis for reasons other than other RCDs, cell necrosis, or activation of immune cells. Nonetheless, we are aware that this result does not provide direct evidence that extracellular HMGB1 originates from necroptosis in sepsis. Various mechanisms of extracellular HMGB1 secretion and complex pathways of necroptosis as well as possibility of HMGB1-mediated necroptosis activation limits definite conclusions^39^. Additional investigations, including single cell study and gene expression analysis are necessary to confirm this speculation.

There are several limitations associated with our study. First, our study included patients referred from a single institution. Thus, the results may not be generalized to other settings or hospitals. Second, based on our study protocol, enrollment of a study patient and sampling of blood were completed within 24 h of ICU admission and 48 h of enrollment, respectively. Therefore, patients with high severity and early mortality may not have been included in the study. Interpretation of results requires caution given that there may be a selection bias. Third, the validation cohort of our study was drawn from a second prospective registry. Therefore, additional confirmatory cohort may be required.

In summary, plasma levels of HMGB1 were associated with severity and prognosis of patient with sepsis. Plasma HMGB1 was correlated with that of RIPK3 and MLKL suggesting its association with necroptosis.

## Data availability

The data that support the findings of this study are available on request from the corresponding author. The data are not publicly available due to privacy or ethical restrictions.

## Supplementary Information

Supplementary information.

## Abbreviations

APACHE II: Acute physiology, age, chronic health evaluation II
DAMP: Damage-associated molecular pattern
HMGB1: High-mobility group box-1
ICU: Intensive care unit
MLKL: Mixed lineage kinase domain-like protein
RCD: Regulated cell death
RIPK3: Receptor-interacting protein kinase-3
SAPS3: Simplified acute physiology score 3
SOFA: Sequential organ failure assessment
