# Supplementary material for: Association of plasma level of high-mobility group box-1 with necroptosis and sepsis outcomes
Source: Sci Rep. 2021 May 4;11:9512. doi: 10.1038/s41598-021-88970-6 (PMC8097071; doi:10.1038/s41598-021-88970-6)
Supplement: Supplementary file 1 — Supplementary information. [file 41598_2021_88970_MOESM1_ESM.docx]

**Association of plasma level of high-mobility group box-1 with necroptosis and sepsis outcomes**

Hongseok Yoo, Yunjoo Im, Ryoung-Eun Ko, Jin Young Lee, Junseon Park, Kyeongman Jeon

**Supplementary Figure S6.** Correlations between plasma level of HMGB1 and RIPK3 (A) and MLKL (B) in validation cohort (n = 77) [RIPK3; slope 195.9 (95% CI: 169.1–222.6), R^2^: 0.7394 (*P* < 0.001), Pearson’s: 0.892 (*P* < 0.001), Spearman’s rho: 0.860 (*P* < 0.001)] [MLKL; slope 0.3554 (95% CI: 0.2863–0.4244), R^2^: 0.5940 (*P* < 0.001), Pearson’s: 0.808 (*P* < 0.001), Spearman’s rho: 0.772 (*P* < 0.001)].

**
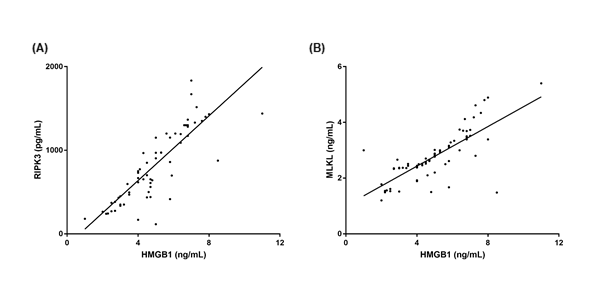
**
